# Automation and standardization of subject-specific region-of-interest segmentation for investigation of diffusion imaging in clinical populations

**DOI:** 10.1371/journal.pone.0268233

**Published:** 2022-12-08

**Authors:** Adriana M. Azor, David J. Sharp, Amy E. Jolly, Niall J. Bourke, Peter J. Hellyer

**Affiliations:** 1 Computational, Cognitive and Clinical Neuroimaging Laboratory, Hammersmith Hospital, London, United Kingdom; 2 Dyson School of Design Engineering, Imperial College London, South Kensington Campus, London, United Kingdom; 3 The Royal British Legion, Centre for Blast Injury Studies, Imperial College London, South Kensington Campus, London, United Kingdom; 4 Care Research & Technology Centre, UK Dementia Research Institute, Imperial College London, London, United Kingdom; 5 Centre for Neuroimaging Sciences, King’s College London, London, United Kingdom; UNITED STATES

## Abstract

Diffusion weighted imaging (DWI) is key in clinical neuroimaging studies. In recent years, DWI has undergone rapid evolution and increasing applications. Diffusion magnetic resonance imaging (dMRI) is widely used to analyse group-level differences in white matter (WM), but suffers from limitations that can be particularly impactful in clinical groups where 1) structural abnormalities may increase erroneous inter-subject registration and 2) subtle differences in WM microstructure between individuals can be missed. It also lacks standardization protocols for analyses at the subject level. Region of Interest (ROI) analyses in native diffusion space can help overcome these challenges, with manual segmentation still used as the gold standard. However, robust automated approaches for the analysis of ROI-extracted native diffusion characteristics are limited. Subject-Specific Diffusion Segmentation (SSDS) is an automated pipeline that uses pre-existing imaging analysis methods to carry out WM investigations in native diffusion space, while overcoming the need to interpolate diffusion images and using an intermediate T1 image to limit registration errors and guide segmentation. SSDS is validated in a cohort of healthy subjects scanned three times to derive test-retest reliability measures and compared to other methods, namely manual segmentation and tract-based spatial statistics as an example of group-level method. The performance of the pipeline is further tested in a clinical population of patients with traumatic brain injury and structural abnormalities. Mean FA values obtained from SSDS showed high test-retest and were similar to FA values estimated from the manual segmentation of the same ROIs (p-value > 0.1). The average dice similarity coefficients (DSCs) comparing results from SSDS and manual segmentations was 0.8 ± 0.1. Case studies of TBI patients showed robustness to the presence of significant structural abnormalities, indicating its potential clinical application in the identification and diagnosis of WM abnormalities. Further recommendation is given regarding the tracts used with SSDS.

## Introduction

Diffusion magnetic resonance imaging (dMRI) is widely used to investigate white matter (WM) microstructure [1, 2]. The different analysis techniques used in dMRI studies have proved extremely useful for determining brain structural connectivity as well as for the quantification of WM abnormalities in a wide range of disorders [3–5]. Region of Interest (ROI) approaches are the most popular type of analysis and these are often combined with voxel-based analyses (VBA) carried out on images registered into standard space [6]. In studies where ROIs need to be defined on native diffusion space, these are often drawn manually onto parametric maps such as fractional anisotropy (FA) maps. This approach is time-consuming, requires specialist anatomical knowledge, and can lead to biased estimation of diffusion metrics [7]. Some techniques, such as the white matter parcellation map (WMPM) are used to guide the manual ROI segmentation and overcome some of the limitations such as hypothesis-driven investigation, or the time-consuming aspect of ROI drawing [8]. In contrast, group-level analysis relies on the combined analysis of diffusion images registered into group space [7], which is automated, highly specific and reproducible. Different DWI normalization and registration techniques have been used and tested such as large deformation diffeomorphic metric mapping [9] or tracts-based spatial statistics [10]. However, to overcome the errors arising from interpolation of the data, methods such as TBSS, for example, can become conservative (i.e. measuring FA at the centre of the tract) and can be vulnerable to inaccuracies introduced by the registration technique in different cases: when structural abnormalities are present, where differences between groups are very subtle or region-specific (e.g. different injury biomechanism), or in the cases in which analysis at the individual level becomes essential [11–13]. In studies on populations with TBI, for example, DTI techniques are sensitive to WM damage at the group level, with insufficient evidence of diffusivity changes at the individual level [14]. Methods deriving individual measures from group-level analyses have been used, but suffer from the same technical limitations [15]. The gold standard of diffusion investigations at the individual level remains manual segmentation of ROIs, with this technique being useful when structural abnormalities (lesions, tumors, etc..) are present, and where tract-based analyses, or registration of the diffusion image become either very challenging or impossible [16]. Fully-automated template based segmentation still require normalization of the DWI data [8], and although it has previously been established that although interpolating DWI images by up-sampling can improve anatomical details, the conventional methods used for the interpolations can impact the results, more so in anatomically atypical brains [17], further highlighting the need for a standardized ROI-segmentation pipeline in native space [16].

We have therefore developed a method that generates robust and repeatable segmentations at the level of the individual diffusion image, while replicating the accuracy of manual ROI segmentation in diffusion space and maintaining the specificity and reproducibility of group-level analysis. The Subject-Specific Diffusion Segmentation (SSDS) pipeline uses existing DTI analysis tools and predefined atlas-based ROIs. Through back-projection of a template into native T1 space, and the use of the anatomical image as a guide for registration of a high-resolution atlas to the diffusion image and its segmentation, SSDS limits errors due to interpolating lower resolution diffusion images [18], and limits the manipulation of raw diffusion data (mainly movement correction and tensor fittings). Through a combination of registration, erosion and masking, our pipeline requires minimal intervention, and generates DWI-specific ROIs on parametric map with high reproducibility and the accuracy of manual segmentation. The pipeline can be used to segment whole-brain WM maps, specific WM tracts, boundaries, or any ROI based on a pre-existing atlas or study mask, and overcomes the challenges presented by structural abnormalities such as lesions or tumours. Moreover, by eliminating the need for parameter changes, and with pre-calculated reliability and reproducibility, SSDS can be used as a homogenous methodology for easier reproducibility across studies, which is an important limitation of individual ROI-based DTI studies to date. SSDS yields mean diffusion metric values for a given ROI, or, when a single mean value does not reflect complete or adequate information about a region that may present heterogeneous abnormalities, SSDS can generate the distribution of values across the ROI. The novelty of the pipeline is in producing accurate ROIs in native diffusion space based on the underlying anatomy through a fully automated methodology, and while limiting interpolation of diffusion data to better and unbiasedly reflect changes in WM microstructure. The T1 image is used as a mid-point registration reference and a guide of segmentation based on the underlying anatomy. SSDS can be used for the following cases 1) The clinical group shows neuroanatomical abnormalities which might be challenging for the between-subject transformation/registration required in group-level analyses, 2) The WM injury difference between groups is more subtle and/or not homogenously distributed across the skeleton and specific subregions, such as the boundary, should be investigated, and 3) The work included individual clinical investigations and/or case studies compared to typical group distributions.

The pipeline was validated using a control group scanned three times, and 47 tracts that are part of the JHU WM atlas (https://identifiers.org/neurovault.collection:264). We present the performance of our registration pipeline and choose FA as an example of diffusion metrics to show it can be estimated from segmented tracts in individual space with high reproducibility and with the accuracy of manual segmentation, both in terms of dice similarity coefficients (DSCs) and accuracy of FA values. We also apply the pipeline to a clinical group of patients with moderate/severe traumatic brain injury (TBI) who present varying levels of focal and/or diffuse axonal injury and atypical structural scans. This type of brain injury can present challenges to traditional approaches of tract segmentation due to structural abnormalities, and we show that SSDS performs well in the estimation of tract FA in this situation. We also present further considerations and possible applications for use of this pipeline.

## Methods: Subject-specific diffusion segmentation

### Participants

Written informed consent was obtained for All participants in accordance with the Declaration of Helsinki. The studies were approved by the West London and GTAC Research Ethics Committee (14/LO/0067, 13/LO/1678, 14/LO/1998) (Table 1). Healthy controls were scanned three times, at three separate visits at the same scanner. The fully automated pipeline was independently tested on all three scans for 17 subjects. The clinical population was scanned once. The same scanner was used for both cohorts.

**Table 1:** Cohort demographics. Demographics of patients and healthy controls used in the validation and testing of the SDSS pipeline.

	n (count)	Male: Female (ratio)	Mean Age ± SD (years)	Presence of Lesions
**Healthy Controls**				
	17	11:6	32.1± 4.2	NA
**Clinical Population**				
Controls	10	5:5	32.8±6.3	NA
TBI patients	19	15:4	43±9.16	52%

### Imaging acquisition

Scanning used a 3T Siemens Magnetom Verio Syngo with a 32-channel head coil. Scanning session for each participant generated a structural high-resolution image T1-weighted MPRAGE image (106 1-mm thick transverse slices, TR = 2300ms, TE = 2.98ms, FA = 9°, inplane resolution = 1x1mm, matrix size = 256x256, field of view = 25.6cmx25.6cm), a diffusion-weighted image (64 directions, b = 1000s/mm^2^, 4 x b0 = 0s/mm^2^, TE/TR = 103/9500ms, 64 contiguous slices, FoV = 256mm, voxel size = 2mm^3^) and a T2 fluid-attenuated inversion recovery (FLAIR) image for lesion identification. The b0 volume used consequently is an average.

### Imaging analysis

**Fig 1:**
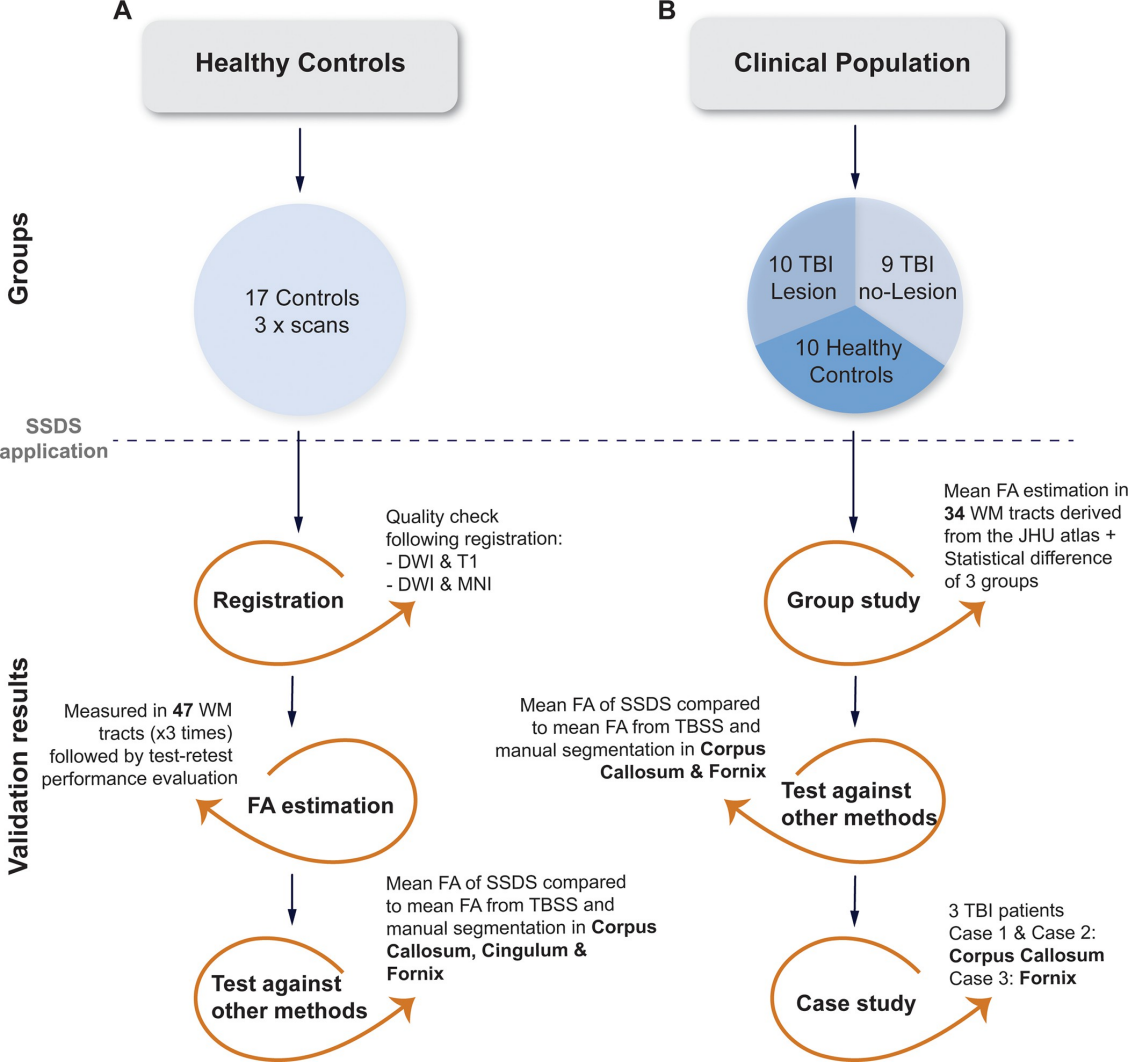
Overview of the validation steps and the results presented in the current study. Analyses on the two cohorts were done separately. The first validations were carried out on the healthy controls (scanned three times for test-retest validation). We present results from the different registration steps, estimation of FA values and test-retest measures, as well as a comparison to other methods. The clinical population (scanned once on the same scanner as the control population) was analyzed first in a group-level study by comparing individually obtained ROI measures, then the results of SSDS were compared to other methods. Finally, three subjects were chosen for case study comparative analyses.

Development and validation of the pipeline were carried out on two separate cohorts: a healthy control group (3 scans for 17 individuals) and a clinical group comprising 3 subgroups: TBI with lesions, TBI without lesions, healthy controls (Fig 1).

#### Overview of SSDS

The requirements for running the Subject-Specific Diffusion Segmentation (SDSS) pipeline are for each subject: a preprocessed DTI image, a T1 image, a gradient field map image (preferred but not required), a standard template and a set of atlas-based tracts or ROIs.

**Fig 2:**
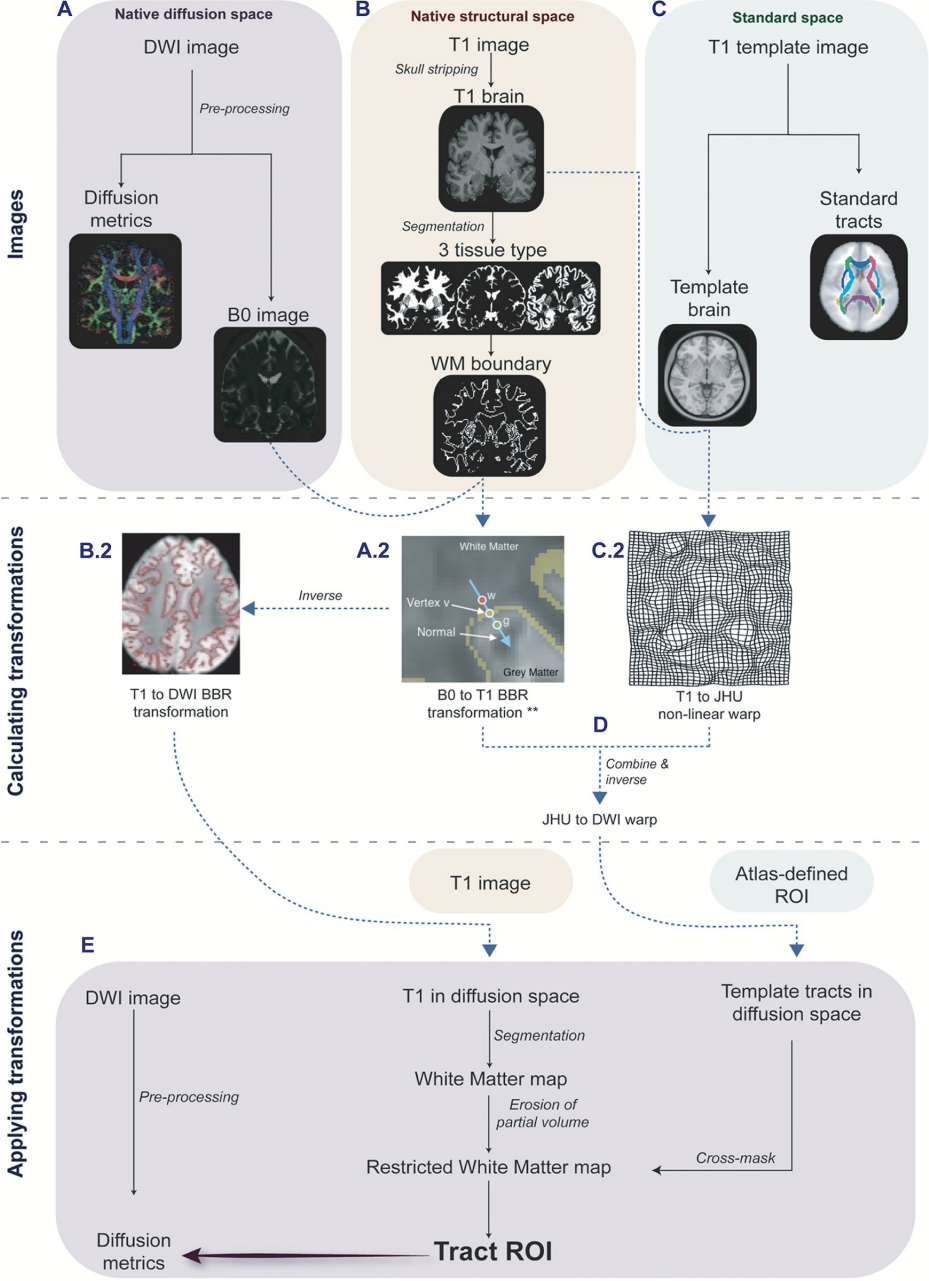
Overview of the pipeline. DWI images are pre-processed individually. 3D T1-weighted images are segmented, the mask of the WM boundary is estimated and used for a BBR of the diffusion image to the T1 image. Non-linear registration is estimated to move the T1 image to a pre-defined template space. The BBR matrix and the non-linear warp are then combined and reversed to estimate a transformation from the standard template to the individual diffusion image. The reverse BBR matrix is applied to the original T1-image, resulting in an inter-modality registration of the T1 to the DWI image. In the last steps, once all three images are in DWI space, the T1-imaged is segmented, the WM map is then used for cross-masking pre-defined ROIs which have been moved to the DWI image. ** image adapted from [19].

To briefly summarize the different steps included in the pipeline (Fig 2):

- DWI images are preprocessed, and a tensor is fitted at each voxel.
- The T1 image is segmented, and a boundary mask of the WM is estimated to drive the registration of the b0 volume to the T1 image and derive a transformation matrix.
- Once the T1 image has been registered in diffusion space, it can be segmented into the difference tissue types to estimate the WM map which will be used to restrict the ROIs to the underlying anatomy.
- Nonlinear warp is estimated by transforming the T1 image to the MNI152 1mm space. The non-linear warp and the BBR-derived matrix are concatenated and inversed to use in a one-step transformation of the ROI tracts from MNI152 1mm space to the individual native space.
- Tracts are then thresholded and masked using the estimated WM map. The resulting binary masks can be used to calculate diffusivity measures from the tracts in individual native space.

#### Pre-processing

*DWI images*. The two main artefacts when dealing with diffusion data are head motion and eddy currents from the gradient coil. Images are aligned via registration to the non-diffusion-weighted (b0) volume to correct for these distortions. b0 volume is then extracted for use as a reference image for subsequent registration, skull stripping and generation of a brain mask in diffusion space. DWI images are preprocessed using FSL’s FDT standard preprocessing technique [20] and the brain extraction tool (BET) for skull-stripping [21]. Motion parameters were applied to the DTI b-vector to compensate for the registration performed to correct for eddy current and motion effects. Finally, the diffusion tensor was estimated using the skull stripped DWI image to generate FA maps using the FSL DTIFIT algorithm (weighted least squares approach). The tensor is estimated at each voxel, using a simple least-square fit of the tensor model to the diffusion data. Metrics other than the tensor can also be calculated, such as fiber density and cross-section. For demonstration purposes in this paper, we use Fractional Anisotropy (FA) as the metric of interest [22].

*T1 images*. Brain extraction was performed using FSL-BET, then segmented into different tissue types and corrected for spatial intensity variations using FSL FAST [23]. The WM map is used to generate a map of the WM boundary, using a surface model used subsequently for BBR (Fig 2).

#### Registrations

*T1 to native DWI*. Optimized alignment is achieved by first up sampling the b0 volume through registration to T1 and calculating the inverse of the transformation to move the structural image to the subject’s native diffusion space. The inter-modality, intra-subject registration of the T1 image to the b0 volume is done in four steps. 1) An initial FLIRT pre-alignment (DOF 6, nearest-neighbor interpolation) [24] of the diffusion echo-planar imaging (EPI) sequence to the T1 skull-stripped brain image. 2) A registration of the field map to the T1 skull-stripped brain image. Because EPI causes geometric distortions, adding information from field maps results in a more accurate and efficient registration [25], achieving improvements in the geometry of the image, which was revealed to be an important aspect of accurate registration to the T1 image. If a field map is not available, deformable registration to T1 is enough to correct for EPI distortions. 3) The pre-alignment matrix and the registered field map are then used to guide the BBR of the b0 volume to the T1 image (DOF 6, boundary-based registration). BBR uses a reconstructed mesh along the WM boundary of the T1 image, and drives alignment to the b0 volume by spatially aligning the vertices of the mesh with the intensity gradient across the WM boundary [26]. This registration does not require the target image to have high quality as long as grey and white matter are differentiated. 4) The inverse of the output of this transformation is calculated. This is done to preserve the diffusion image in its native space and avoid any interpolation of the date for best estimation of diffusion metrics following segmentation of ROIs. 5) the final transformation matrix is applied to the T1 image using the b0 volume as a reference. The two main outputs of these registrations are a) the subject’s T1 image down-sampled and registered to the subject’s b0 volume (i.e., the T1 image aligned to the DTI image), b) a subject-specific matrix for this transformation which will be subsequently required to drive the registration of the high-resolution ROIs.

***Standard template to native DWI*.** Next, the standard template of interest was registered to the b0 volume. In this case, our regions of interest (ROIs) are derived from the JHU WM atlas. We used the MNI152 1mm standard-space T1-weighted average structural template and estimated a subject-specific warp field of the transformation. Optimized alignment is achieved in two main steps. 1) Registration of the T1 structural image to MNI152 1mm template through a non-linear warp (linear, DOF 6, nearest-neighbor interpolation, followed by non-linear sum-of-squared differences) and 2) Combining the non-linear warp and the BBR matrix and inversing the concatenated transformation to obtain a one-step non-linear transformation of diffusion image to the high-resolution template. Non-linear registration from native diffusion space to standard space is a two-step process and uses the T1 image as a mid-point reference. The final warp field will be the equivalent of the inverse of the following transformations: diffusion to T1 linear BBR + T1 to standard template non-linear transformation. The main output is a subject-specific transformation field to accurately move ROIs defined through the standard template to the b0 volume (i.e., the MNI152 1mm T1-weighted standard template aligned to the DTI image).

#### Segmentations

*Whole tract segmentation*. Restriction of tracts to the underlying WM anatomy helps avoid partial volume effects and improves the estimation of the tracts in DWI space [27]. Segmentation is achieved in six steps. 1) After registration to the b0 volume, the T1 image is segmented. Even with a lower resolution, the segmentation of the T1 image yields more accurate results than the transformation of segmented maps. 2) To optimize exclusion of partial volume voxels the WM map is thresholded to exclude all boundary voxels with values below 0.8 (range 0–1) and binarized. 3) 47 tracts were selected from the ICBM-DTI-81 white-matter labels atlas [28]. 4) The tracts are initially eroded (single voxel, 3x3x3 box centered on target voxel) to limit overlap with partial volume voxels. 5) Using the previously estimated warp field, the 47 WM tracts are then warped to subject-specific diffusion space to guide the segmentation of ROIs. 6.a) After registration to the b0 volume, each tract is cross masked with the estimated WM map and further thresholded and binarized to ensure exclusion of boundary voxels with partial volume errors (threshold 0.8, range 0–1). 6.b) For a more conservative segmentation, a second erosion can be added to the WM map. However, this did not improve FA estimation, and excluded relevant spatial details, and was therefore deemed unnecessary.

**Fig 3:**
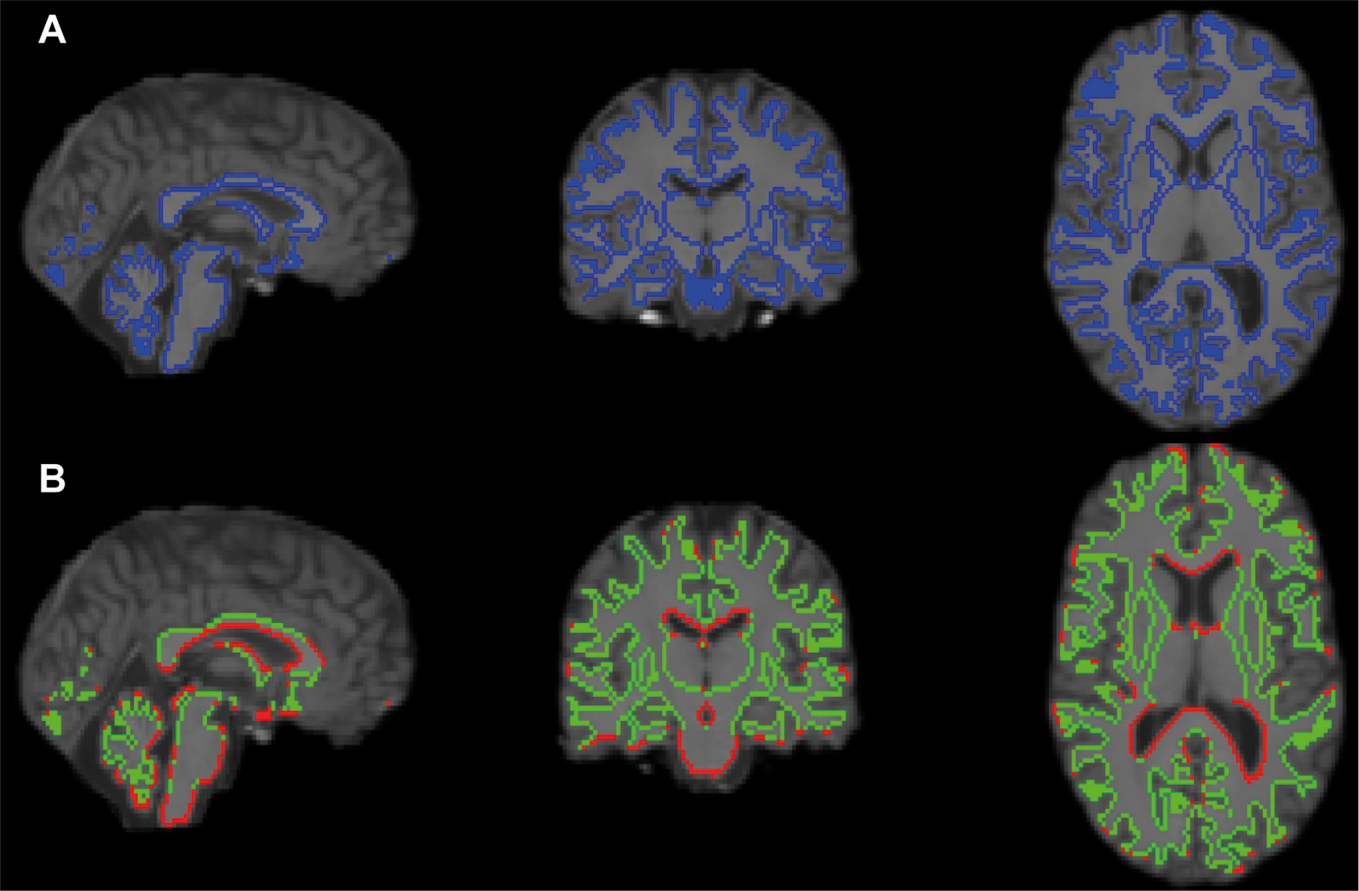
Example of boundary segmentation. A) Boundary of the WM. The blue line is the binary mask. The T1 image is in native diffusion space. B) Boundary of the WM/CSF and WM/GM. The red line is the mask of the WM/CSF boundary and the green line is the mask of the WM/GM boundary. The T1 image is in native diffusion space.

*Boundary segmentation*. To segment the boundaries of the WM, the T1 image is segmented into 3 tissue-type after registration to the B0 image. Once segmented, the WM map is thresholded and binarized, and can then be eroded from all inner voxels to give a binary mask of the entire WM. Even with a lower resolution, the segmentation of the T1 image yields more accurate results than the transformation of segmented maps. The map of the CSF is then dilated and cross-masked with the whole-brain WM boundary map to create the map of the WM/CSF boundary, and this mask if subtracted from the whole-brain WM boundary map to create the map of the WM/GM boundary (Fig 3). Cross masking boundary mask and whole tract mask will then yield GM/WM and CSF/WM boundary mask for each ROI.

#### Validation

For validation of the results obtained from the SSDS pipeline, the chosen ROIs were the body, splenium and genu of the corpus callosum, the right and left cingulum and the fornix. Selection of the ROIs to test the performance of the pipeline was done on the basis of size and anatomy for tracts with different reliability in the validation process. Validation included a comparison against manual segmented ROIs, ROIs obtained following skeletonization through the ENIGMA protocol [29], and ROIs obtained from a one-step non-linear registration of a diffusion image to the high-resolution JHU template. Dice similarity coefficients (DSCs) were calculated to compare the overlap of the automated ROI segmentation through SSDS and the manual segmentation.

*Manual ROI segmentation*. Six tracts were manually traced on each parametric map, using scans from the first visit for 15 healthy subjects. Each ROI consisted of multiple slices fully covering the three-dimensional structure. The anatomical structures were defined according to the definitions provided in a standard DTI atlas [8]. The ROIs were chosen to cover a) large and reliable tracts: the body, splenium and genu of the corpus callosum, b) thinner tracts: the right and left cingulum c) small tracts prone to tensor estimation errors: the fornix [30]. ROIs were selected for comparison against SDSS performance.

*Mean FA from ROI following registration to a standard template*. Individual FA maps are registered to the MNI152 standard template using the 1mm FRMIB FA atlas [10]. Following registration to the high-resolution template, mean FA is estimated through pre-defined ROI masks.

*Mean FA from ROI following TBSS skeletonization*. All FA maps were merged and skeletonized using a 0.2 threshold to ensure exclusion of partial volume effect. The 4D image of concatenated FA maps for each participant was subsequently split. Skeletonized FA maps are intersected with ROI masks to get individual mean FA for each ROI [10].

### Exemplar applications

#### Group-level comparison: TBI vs healthy controls

The application of the pipeline to a clinical population included three groups: 10 healthy controls, 10 subjects with moderate/severe TBI and presence of lesions on the brain, and 9 subjects with mild TBI [31] (Fig 1). Of the 47 tracts available in the JHU WM atlas, 34 tracts were included in this analysis as part of the validation process, based on the results of the test-retest and coefficient of variation that indicated the most reliable tracts for the analysis. Mean FA and histogram distributions of FA values for each tract were extracted. For further validation of the pipeline in a clinical population, 4 of the 34 tracts from the JHU atlas were selected for manual segmentation and skeletonized FA estimation following TBSS. Tracts were chosen to include different sizes and reliability. ROIs included were the three parts of the corpus callosum and the fornix.

#### Case studies

Three cases of severe TBI were analyzed separately. Each case included segmentation of a single tract and comparison across the four different methods: SSDS, manual segmentation, ENIGMA-DTI protocol [29] and masking from a high-resolution template.

### Statistical analysis

Statistical analyses were performed using Rstudio v. 3.5.1. Test-retest reliability was assessed using the following three measures: intraclass correlation [32], coefficient of variation and correlation of measures across visits. Tracts deemed unreliable for use in the SSDS pipeline were excluded based on high intra-subject variation (>5%).

We first used a Tukey non-additivity test on the results obtained from the first visit, such as our two-factor factorial design tracts*subject = 47*17, with n = 1 replicate for each tract*subject combination. We then ran a two-way mixed single measure consistency Intra-class correlation (ICC), with a design of tracts*subject*3visits (47 tracts and 48 raters per tract). This was followed by a 2-level bootstrap with replacement, with 10,000 for our 47*48 design. Following each replication, ICC is estimated to generate a bootstrapped confidence interval of 95%. Coefficient of variation (COV) was estimated ((SD/mean)*100) to assess intra-subject variability of FA and tract size measures across all three visits. Initially, a mean FA value and a tract size (voxel count) were estimated for each tract, across all 16 subjects and three visits. For each subject, a COV was estimated for every tract across the three visits (i.e. one measure per subject per tract). An average of all 16 COV measures was then calculated for every tract. Lastly, Pearson’s correlation coefficients, including all tracts and all subjects were estimated for pairwise comparison across visits. Dice similarity coefficients (DSCs) was calculated to determine the similarity and spatial overlap of the manual segmentation and the ROIs resulting from SSDS.

## Results

### Registration

**Fig 4:**
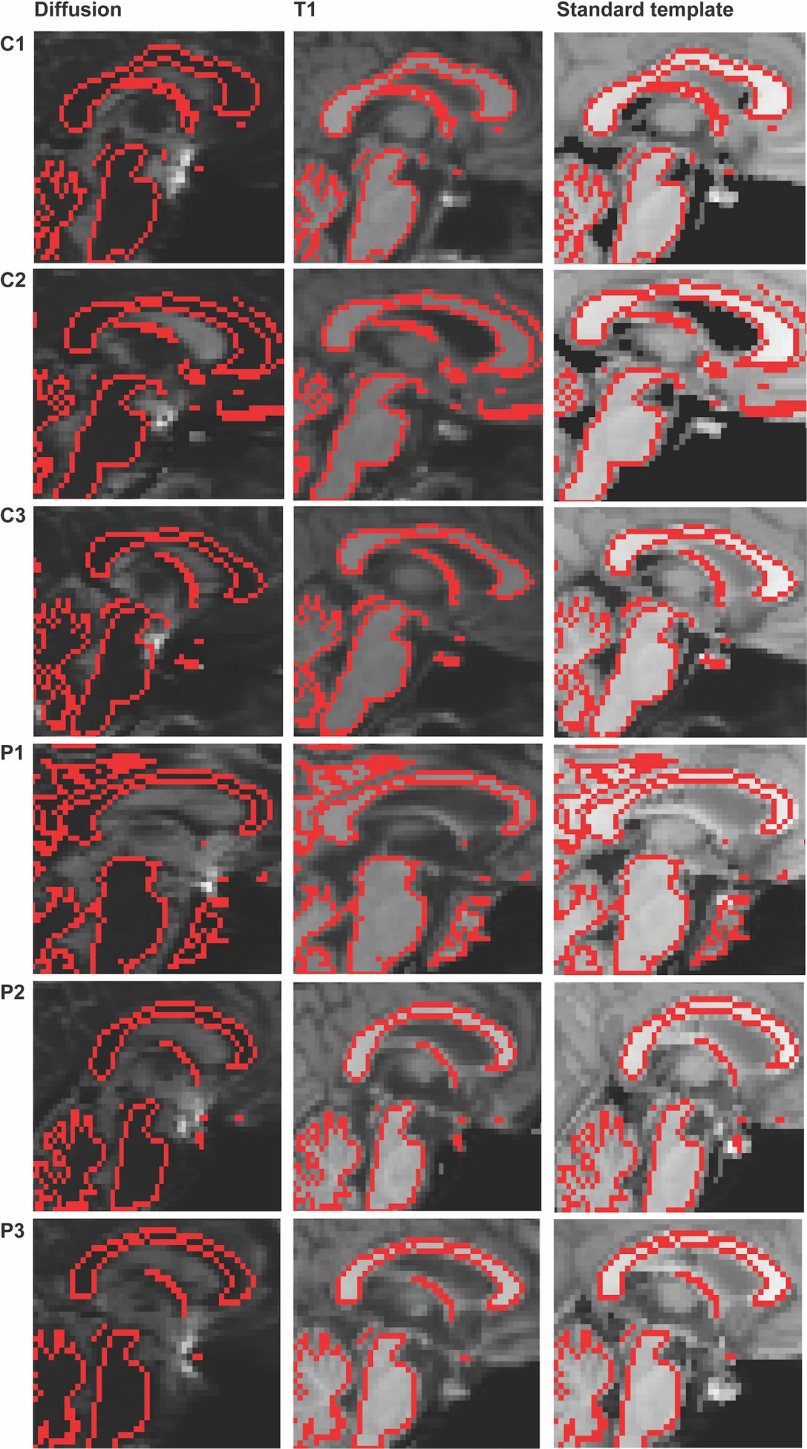
Examples of individual registration performance. Registration of T1 to diffusion image and of standard template to diffusion image on 3 controls (C1, C2, C3) and 3 TBI patients (P1, P2, P3). The mask of the WM boundary (red) resulting from the segmentation of the T1 image in DWI space is used to indicate voxel-wise correspondence among all three images and the accurate structural overlay.

We used cross-modality registration between a subject’s T1 and DTI images, which allowed accurate individual level analysis of tract ROIs back-projected into individual space. Individual T1 images were registered to the lower resolution diffusion image using linear BBR (EPI to T1 image). For inter-modality registration, results obtained using BBR were more accurate than the alternative linear (FLIRT) and non-linear (FNIRT) methods. The MNI152 higher resolution standard template was registered to the native DTI image using non-linear registration to T1 and the precalculated BBR matrix. For visual quality check, a WM boundary mask was defined in the T1 image registered to the b0 volume. The boundary mask was overlaid on the 3 images following registration in DWI space (diffusion, T1 JHU template). This visual assessment of the registration shows accurate alignment of the three images in 3 healthy subjects (C1,C2,C3) and 3 subjects with moderate/severe TBI (P1,P2,P3) (Fig 4). The overlay of the WM boundary mask shows a voxel-wise correspondence among all three images for all cases after being moved to the individual subject diffusion space.

### Tract segmentation and estimation of fractional anisotropy

**Fig 5:**
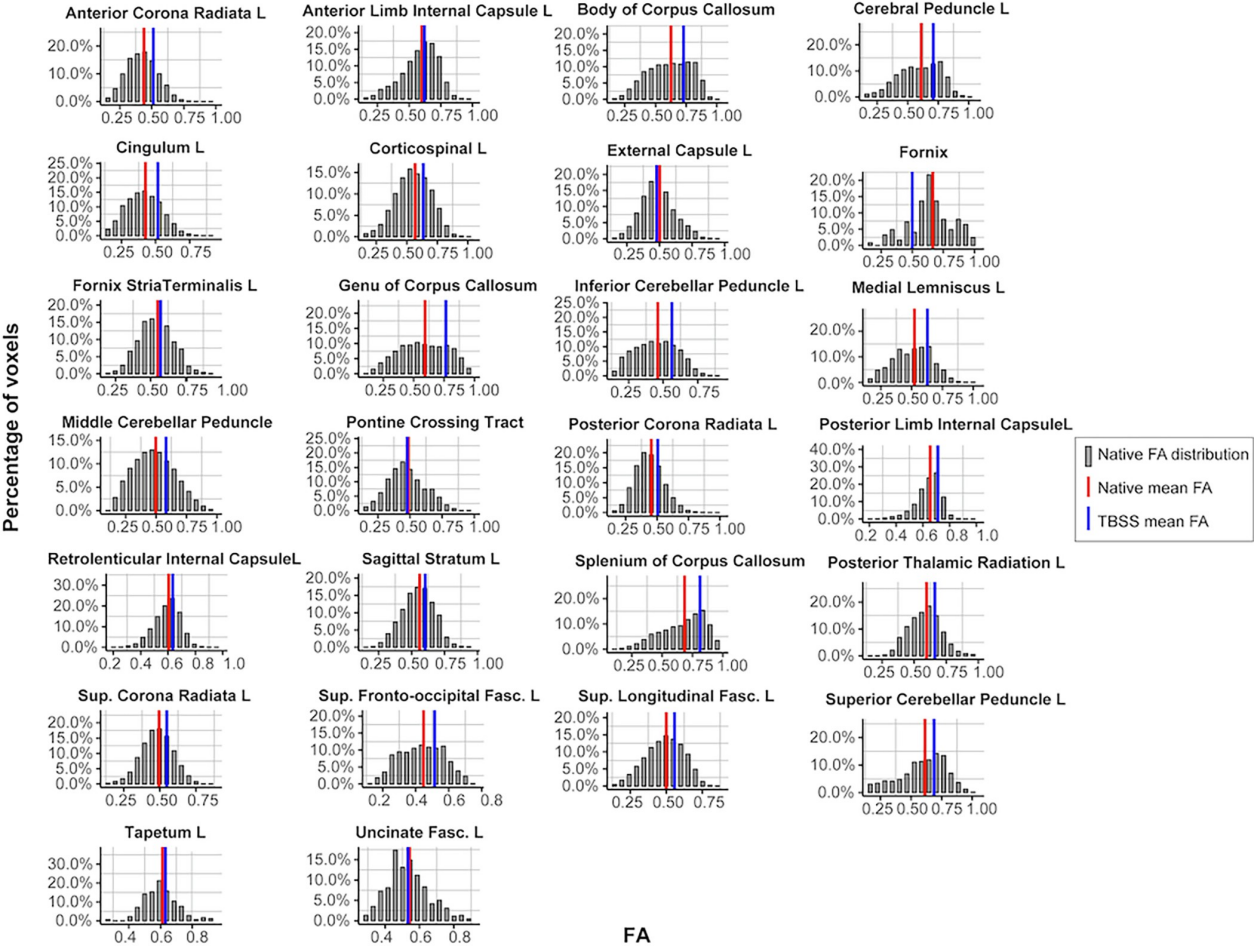
Histogram of FA distribution. The histogram represents the frequency distribution of FA values for every voxel in a given tract following segmentation of the whole tract in native diffusion space. Only left hemisphere tracts are included. The native mean FA is plotted on the distribution (red).

47 tracts were segmented to assess the performance of the pipeline. Visual quality assessment was carried out by overlaying the segmented tracts on the parametric FA map and assessing voxel-wise correspondence. No segmentation needed manual intervention for correction (example of segmentation in S1 Fig). For every ROI, histogram of FA values across the tract can be extracted. The distribution of FA values and the single mean value for a subset representation of 26 tracts were extracted and plotted for individual subjects (Fig 5).

### Test-retest reliability

**Fig 6:**
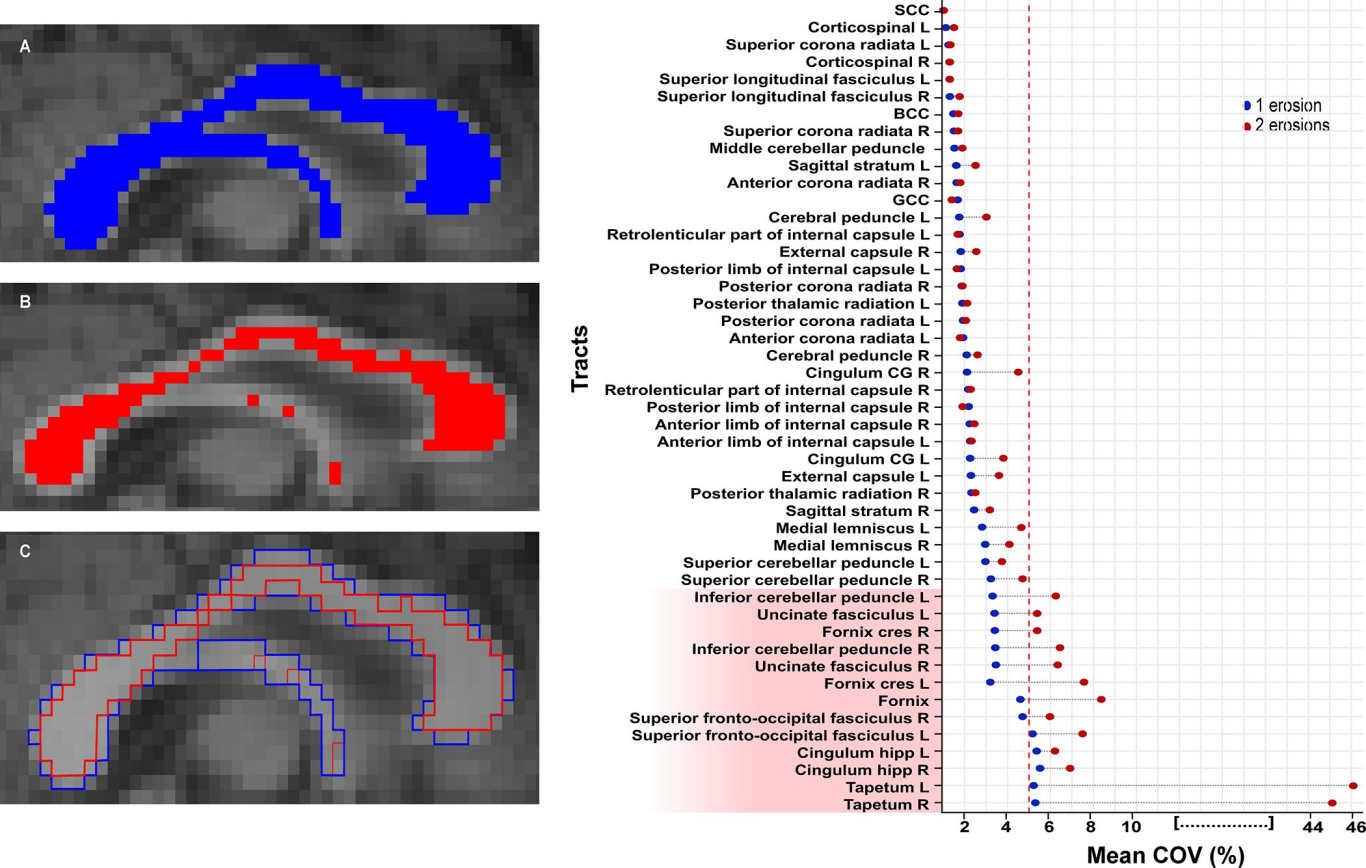
Tracts erosion and coefficient of variation. **(Left)** Direct comparison of segmentation and voxel exclusion using (A) an n-pass filter of 1 or (B) an n-pass filter of 2 in the fornix and the three parts of the corpus callosum, and (C) the difference between the two erosion filters. **(Right)** Mean intra-subject COV per tract values following segmentation of 47 tracts using an n-pass filter of 1 (blue) or 2 (red). Cut-off (red dotted line) is at 5% variation for any of the two erosions (if variation of either 1 or 2 erosions is higher than 5%). Tracts highlighted in red are rejected. BCC = body of corpus callosum, GCC = genu of corpus callosum, SCC = splenium of corpus callosum.

Tract diffusion measures generated using SSDS showed high test-retest reliability. Tract FA was generally very similar across the three visits assessed: average COV was 2.7% ± 1.5% with 1-erosion parameter, and 5.1% ± 8.6% with a 2-erosion parameter (Fig 6). 13 tracts had a COV greater than 5% in either erosion parameters used: the fornix, superior fronto-occipital fasciculus bilaterally, hippocampal cingulum bundle bilaterally, the inferior cerebellar peduncle bilaterally, the uncinate fasciculus bilaterally, the fornix crescent bilaterally, and the tapetum bilaterally (Fig 6). These tracts with higher COV tended to be smaller, with a strong correlation seen between the size of the tract, based on voxel count, and the COV (correlation coefficient r = -0.64, p < 0.001) (Table 2). In subsequent analysis, we omit these tracts from the SSDS pipeline. However, we used the fornix for visualisation and comparison purposes, but caution is advised when segmenting the tracts mentioned above. For conservative measures, we suggest the use of a 2-erosion parameters. However, we use 1 erosion is subsequent analyses to include important anatomical information from the whole tract and reduce variability (Fig 6A).

**Table 2:** Test-retest results.

	ICC				Average COV		Correlation pairwise (FA)		
Category	Lower	Upper	Result	F	Intra-subject	Intra-tract	Visits 1&2	Visits 1&3	Visits 2&3
**Values**	0.742	0.870	0.808	203	2.61%	0.92%	0.95	0.94	0.94
**P-value**	<0.001				NA		<0.001	<0.001	<0.001
**Boot ICC**	0.714–0.838				NA		NA	NA	NA

Reported results for mean intraclass correlation test (ICC), average coefficient of variation (COV) for within tracts and within-subject measurements, and pairwise FA correlation. ICC, COV and correlation analyses were carried on all 47 tracts, the average is reported. Boot ICC are results of the confidence interval following a bootstrap test on our data.

**Fig 7:**
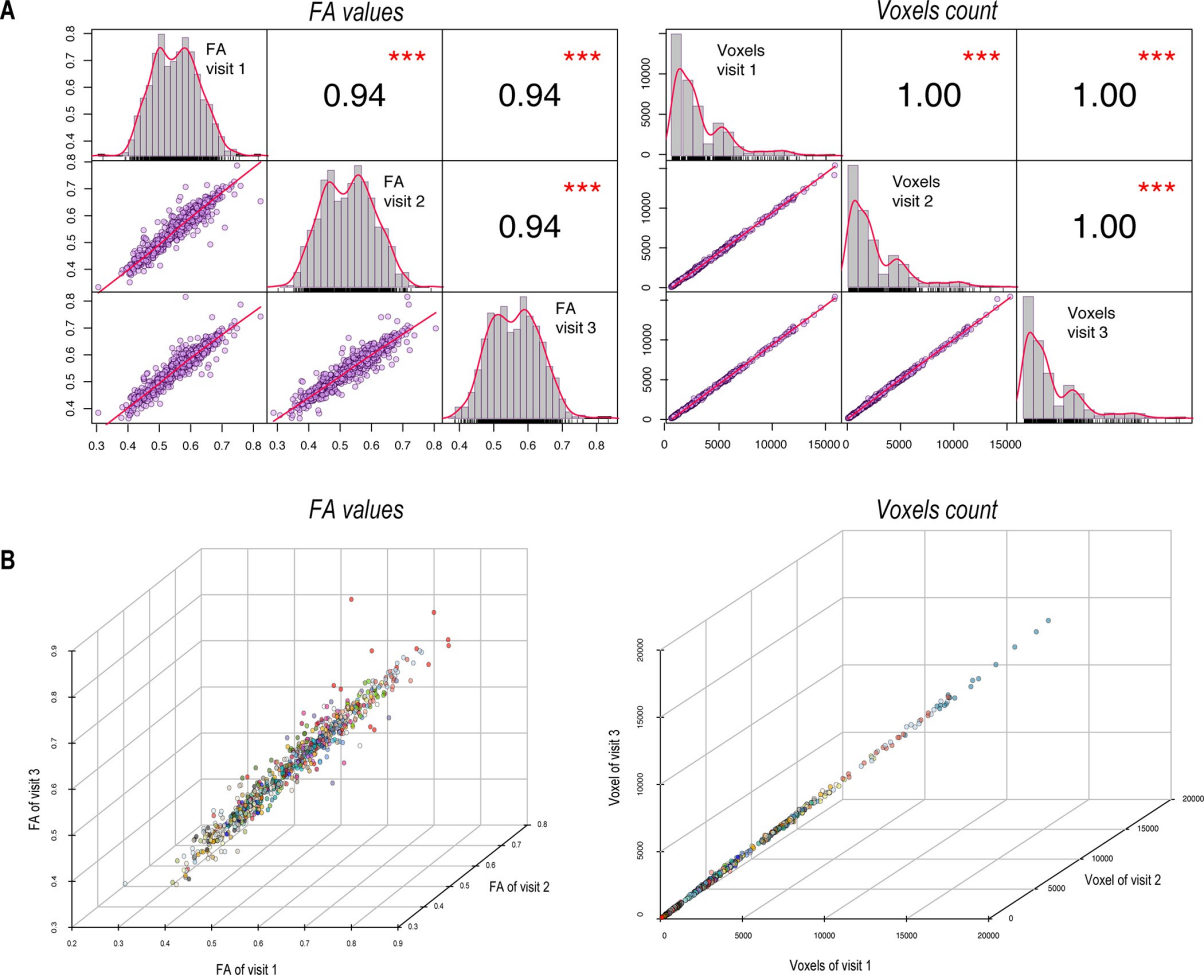
Test-retest correlation across subjects and visits. **(A)** Pairwise correlation matrix of mean FA values and voxel count per tract per subject. Upper right values represent correlation coefficients r, histogram represent mean FA and voxel count distributions and scatterplot represent pairwise correlation of data points. P-values: ***<0.01. **(B)** 3D correlation plot of mean FA value and number of voxels for all three visits per tract, per subject. Each color represents a different tract, for all 47 tracts of the JHU atlas.

To further demonstrate reliability and accuracy, repeated segmentations of each tract (intra-subject, inter-visit) produced segmentations with a similar number of voxels (correlation coefficient r = 1.0, p < 0.001) and very similar estimations of mean tract FA (correlation coefficient r = 0.94, p < 0.001) in all 17 subjects (Fig 7). Using a two-way mixed single measure consistency ICC, we found a mean value across all tracts and all visits of 0.87 (Table 2), considered excellent on the Cicchetti scale [33].

Repeated segmentations of the whole brain boundary, as well as the boundaries of the WM/CSF and WM/GM also showed high test-retest reliability (correlation plot in S2 Fig). Intra-subject, inter-visit segmentations produced results with a similar number of voxels (correlation coefficient r = 0.99, p < 0.001) and very similar estimations of mean FA (correlation coefficient r = 0.78, p < 0.001) in all 17 subjects.

### SSDS vs other methods

**Fig 8:**
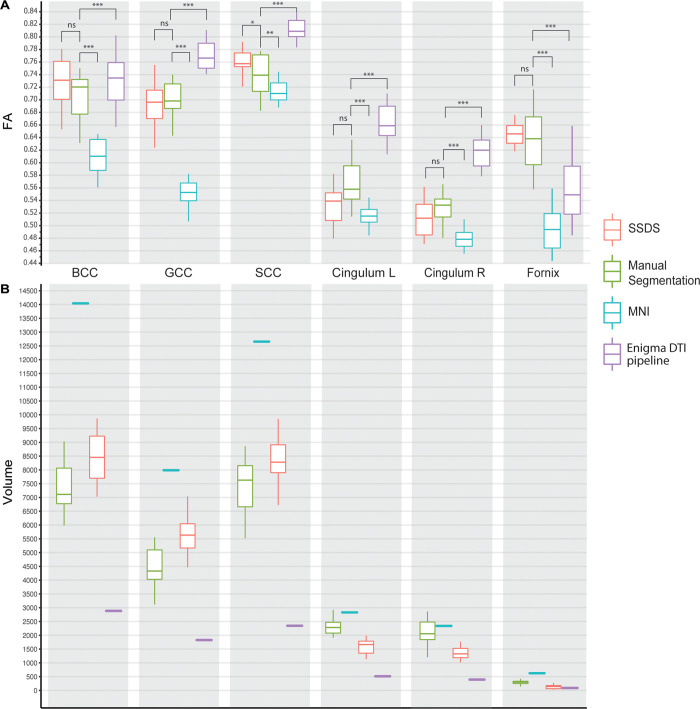
Boxplots of mean FA and volumes of ROIs. Boxplot comparing mean FA values (A) and volume in mm^3^ (B) in n = 15 healthy controls for 4 segmentation methods of the body, genu and splenium of the corpus callosum (BCC, GCC, SCC respectively), fornix and cingulum bilaterally. The four segmentation methods include SSDS, a manual segmentation on the parametric map, crossing ROI with normalized FA in MNI152 template space, and extracting the mean skeletonised FA value of each ROI mask. Although the fornix was omitted from analysis (because of a poor test -retest reliability), we included segmentation of the fornix to illustrate comparisons of the methods on a small tract. Ns = not significant, *p <0.05, **p <0.01, ***p <0.001.

In order to validate the pipeline, we compared results of the SSDS pipeline applied to 6 tracts, chose to represent tracts with different sizes and morphologies: the genu, body and splenium of the corpus callosum, the cingulum bundle left and right, and fornix, to results obtained from a) ROI analysis following manual segmentation on the parametric diffusion map, b) ROI masking following registration to the high resolution MNI152 template, and c) skeletonized mean FA measures of ROI tracts after TBSS and following the ENIGMA DTI pipeline [29] (Fig 8), methods commonly used in clinical investigations of diffusion imaging.

Running a one-way ANOVA for the effect of the method used on the FA value, revealed statistically significant difference on all 6 tracts: the body of the corpus callosum (F = 36.34, p<0.001), the genu of the corpus callosum (F = 78.86, p<0.001), the splenium of the corpus callosum (F = 38.3, p<0.001), the right and left cingulum bundles (F = 22.88, p<0.001 and F = 27.5, p<0.001 respectively) and the fornix (F = 29.33, p<0.001). A comparison of each method pairwise with the results from manual segmentation showed similar mean FA values for SSDS and manual segmentation for 5 tracts, while the mean FA value of the splenium of the corpus callosum is slightly higher using SSDS (t = 1.75, p = 0.046). This reflects high levels of similarity between SSDS and gold-standard manual segmentation. The average DSC value for all tracts comparing ROIs from manual segmentation and SSDS was 0.8 ± 0.1, ranging from 0.5 to 0.97, with the highest DSC average being for the segmentation of the splenium of the corpus callosum (0.81 ± 0.06), and the lowest being for the segmentation of the fornix (0.76 ± 0.13).

In contrast, there was a significant difference between mean FA of the 3 tracts when comparing manual segmentation and mean FA from ROI following registration to a standard template and skeletonized tract (Fig 8). The mean FA extracted following skeletonization and using the ENIGMA DTI pipeline were on average higher than the mean FA values estimated through SSDS which would be expected given that the skeletonization process projects expected peak FA values onto the skeleton while ignoring neighbouring voxels.

Following non-linear registration of the parametric map to the standard template, and before skeletonization, the volume of the tract segmented is on average 1.5 folds higher than the mean values for the corresponding tracts in native diffusion space. This transformation to a higher resolution template includes smoothed voxels and partial volume effect, an effect of registration that is further exacerbated by the lower mean FA values in the corresponding tract (Fig 8A) if all voxels are included to derive the mean value.

After skeletonization and exclusion of the partial volume to limit measurements to the more central part of the tract, and following the ENIGMA DTI pipeline, the volume of the skeletonised ROI is on average 3 times smaller than that of the ROI segmented in native space (Fig 8B), sampling FA values from peak signal voxels by projection of these voxels onto the skeleton, yields a higher mean FA value for the given ROIs. Even when outermost voxels are eroded, the diffusivity measures obtained from SSDS sample a larger volume without being skewed to peak values which are obtained from the skeletonization process. This reflects the limitation of only looking at peak FA values in samples where microstructural differences might not be homogenously distributed across the skeleton.

Comparing reliability for FA values obtained from the different methods (Fig 8A), intra-tract (same tract, different subject) COV for the manual segmentation and TBSS stands at 6.60% and 7.23% respectively, compared to 7.26% for SSDS, which indicates similar variation for SSDS to the methods commonly used.

### Example–TBI patients vs healthy controls

**Fig 9:**
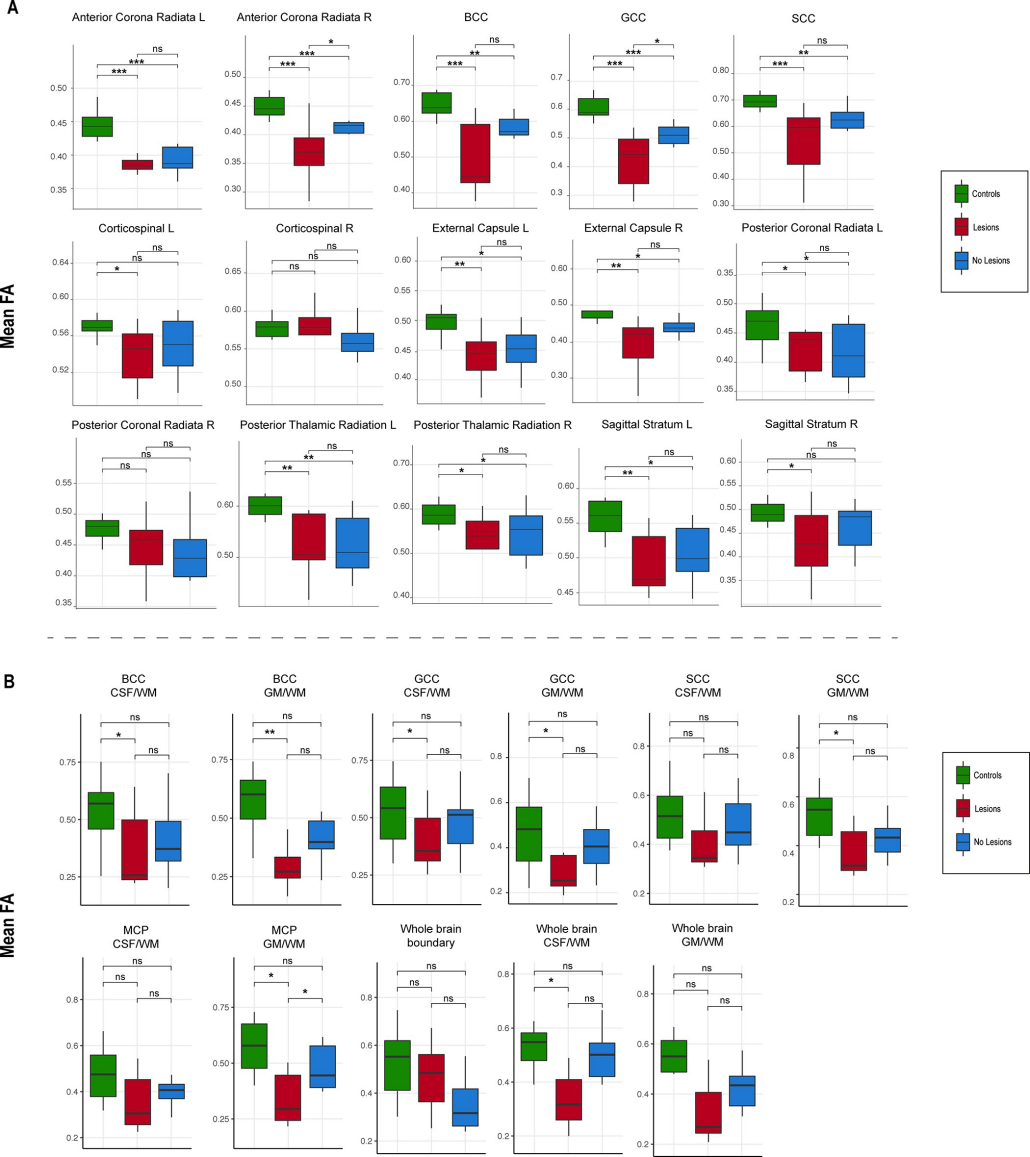
Tract-specific mean FA value. Comparison in 3 groups: healthy controls (green), TBI with lesions (red), and TBI without lesions (blue). A) whole tract segmentation, B) boundary of tracts segmentation. A subset of tracts is considered in this analysis, mainly when at least one of the bilateral tracts showed a difference. Ns = non-significant, BCC = boundary of the corpus callosum, GCC = genu of the corpus callosum, SCC = splenium of the corpus callosum, CSF/WM = boundary of the CSF and the WM, GM/WM = boundary of the GM and the WM. *p<0.05, **p<0.01, ***p<0.001.

As an example of clinical application of SSDS, we looked TBI patients with and without lesions. Mean FA derived from the whole brain WM map was significantly lower in both TBI groups (with and without lesions) compared to the healthy control group (t = 6.59, p<0.001 and t = 5.25, p<0.001 respectively), with the lowest WM mean FA recorded in TBI patients with lesions, but not significantly lower than mean FA of TBI patients with no lesions (respective mean X_1_ = 0.5 ± 0.1 and X_2_ = 0.51 ± 0.08, t = -1.62 p = 0.053). Tract-specific values reveal a decrease in mean FA in the group with lesion compared to the control and no-lesion groups in the right anterior corona radiata, and the genu of the corpus callosum (Fig 9A). The boundary of the WM in the whole brain did not show a significant mean FA decrease in both TBI groups compared to controls. This was also the case in the whole brain WM/GM boundary. However, some tracts did show a difference in mean FA when comparing the controls and the TBI group with lesions. The tracts include the whole brain WM/CSF boundary, the boundaries of the body and the genu of the corpus callosum, the GM/WM boundary of the splenium of the corpus callosum. The GM/WM boundary of the middle cerebellar peduncle showed a significant decrease of the mean FA value compared to both the controls and the TBI with no lesions (Fig 9B).

To demonstrate that SSDS provides robust FA estimates in cases of severe brain injury with structural abnormalities, we applied it to three case studies, and tested it against the other common group-level methods.

**Fig 10:**
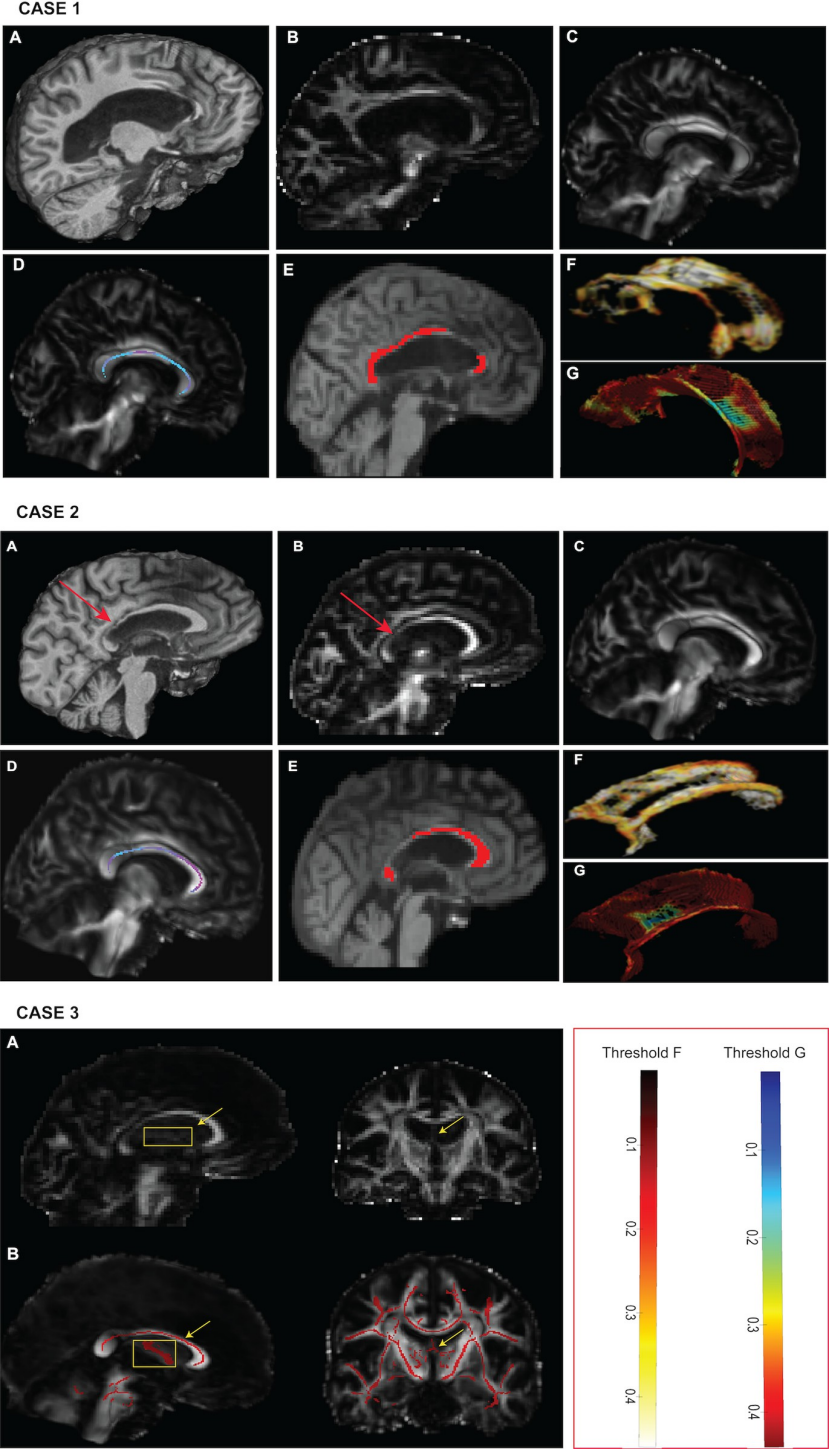
Visual representation of case studies of patients with severe TBI. Cases 1 and 2 show the segmentation of the corpus callosum, and case 3 illustrates the robustness of the pipeline when a signal is not detected on the DWI image. A) native T1 image to illustrate structural abnormality before registration to the diffusion image, B) FA map in native diffusion space, C) FA map following registration to a higher resolution standard template through non-linear alignment, D) TBSS segmentation of corpus callosum and heatmap of FA values, E) SSDS segmentation of the tract, F) 3D rendering results of the segmentation of the Corpus Callosum through SSDS and corresponding heatmap (Threshold F), G) 3D rendering results of the segmentation of the Corpus Callosum through TBSS and corresponding heatmap (Threshold G).

Case 1 shows a patient with loss of volume within the corpus callosum accompanied by ventricular enlargement. Severe damage is seen in the genu of the corpus callosum (Fig 10 -case 1). When assessed in native space, the affected WM region does not form part of the manual segmentation by the rater nor by the automated segmentation of SSDS given the lack of signal from both the T1 and the diffusion image. In contrast, the process of registration to a standard space produces an estimated WM map and a subsequent skeleton within this location if TBSS is performed. Following TBSS estimation of a skeleton at this region, where peak voxels are selected and projected on the skeleton, the mean FA value is calculated as 0.514. Following SSDS the mean FA value for the intact region of the corpus callosum is 0.364, against 0.382 for a manually segmented ROI of the same region.

Case 2 illustrates a similar situation, with thinning of the WM at the junction of the splenium and the body of the corpus callosum. When assessed in individual space, this WM region does not form part of the manual segmentation nor the automated segmentation of SSDS (mean FA value of the segmented the corpus callosum is 0.450 and 0.425 respectively), due to the low-level signal in both the T1 and the FA map at this specific location (Fig 10– case 2 A and B). In contrast, the process of normalization to a standard space (Case 2- C), and enlargement of the tract (as seen in 3.4) produces an estimated WM map and subsequent skeleton within this location. Following TBSS estimation of a skeleton at this region, the mean FA value is calculated as 0.532.

Case 3 illustrates a brain with significant atrophy, resulting in loss of signal from small tracts around the ventricles such as the fornix. For the third case (Fig 10 –Case 3), the rater was unable to segment the fornix directly on the FA map. SSDS also returned an empty mask when trying to segment the fornix. However, registration to a high-resolution template yields an estimated WM signal in this area, and a mean FA value of **0.15**, and the skeletonized tract returned a mean FA value of **0.16**.

Our case studies show that SSDS reflects results from manual segmentation more accurately in individual investigations and is robust to the presence of signal abnormalities on the diffusion scan.

## Discussion

Subject-Specific Diffusion Segmentation (SSDS) combines the spatial accuracy of manual tract segmentation on native parametric maps, with the standardization, automation and reproducibility of group-level approaches. It overcomes the need for time-consuming manual tracing in studies investigating individual changes in ROIs, subtle group-level differences, or in clinical groups with severe anatomical abnormalities in which registration of the diffusion image can yield inaccurate results. SSDS relies on the underlying anatomy by using a T1 structural image to guide both registration and segmentation. The diffusion image is never warped to the high-resolution template. Instead, the T1 image drives the registration of the high-resolution template into the native diffusion space and preserves the anatomical details of the standard templates and the T1 image and the integrity of the diffusion information. For the accurate intra-subject, inter-modality registration, we use a BBR, which is based on the definition of a WM boundary and the difference of intensity across the boundary in both structural and diffusion images. Integrating BBR and the ‘up sampling-combine-inverse’ technique resulted in an accurate registration of both the template and the T1 image in the lower-resolution DWI space in structurally typical as well as atypical brains suffering from anatomical abnormalities. Although SSDS is not the first automated method for measuring ROI-specific in diffusion information [34], most standardized and commonly used pipelines perform analyses in group space. Standardized and publicly available simple protocols with pre-defined test-retest reliability and parameter normalization for more homogenous individual diffusion investigations are still lacking. SSDS uses common registration techniques and predefined atlases to make automated segmentation stable in native diffusion space, maintaining high correspondence with FA values obtained from manual segmentation of ROI, and providing a reproducible pipeline capable of replacing manual segmentation of ROIs on parametric maps in clinical subject-level investigations. The segmentation focuses on areas of the WM tract that is often overlooked. Being able to segment the boundary of certain tracts and divide them into WM–GM boundary vs WM–CSF boundary is relevant in many clinical applications, including but not limited to TBI and autism. SSDS is simple and straightforward. It also sheds the light on the importance of analysis at the subject level, the importance of analysis on native diffusion data, and the importance of specific region of the white matter such as the boundary and the possibility of segmenting those areas with methods other than manual segmentation. One model that seems to deal well with both native diffusion data and the presence of structural abnormalities is the atlas-based Markov random field representation [35]. However, the model focuses much more on crossing fibres, and because it does not rely on the underlying structural data, the anatomical definition of tracts and tracts boundaries and constraint to the WM is less precise.

SSDS shows high test-retest reliability across subjects and visits, for both the FA value calculated and the size of the tracts. Out of the 47 tracts segmented and tested, we eliminate tracts with a COV value higher 5%. The excluded tracts were the fornix, superior fronto-occipital fasciculus bilaterally, hippocampal cingulum bundle bilaterally, the inferior cerebellar peduncle bilaterally, the uncinate fasciculus bilaterally, the fornix crescent bilaterally, and the tapetum bilaterally. The tracts with high variability show a strong negative correlation with size. We also used 1-erosion of tracts for all subsequent analysis. Although adding a second erosion allows for more conservative segmentation, it increases the variation in measured FA and excludes important spatial information from the tracts. SSDS also reflects manual segmentation more accurately than the other methods and shows a high dice similarity coefficients (DSC) score on tracts studied. Like manual segmentation, SSDS retains the size and the morphology of the ROI, while in group-level analysis, spatial normalization of the parametric map to a higher resolution template means warping and smoothing, and normalizing anatomy to achieve voxel-to-voxel correspondence for the subjects studied [2]. Warping can result in errors when moving images of low resolution and poorer contrast (such as DWI) to high template resolution [11]. Smoothing is often used to increase signal-to-noise ratio and compensate for possible misalignment, creating a normal distribution of the data [36]. Although spatial normalization has been shown to preserve micro and macro-structural properties of large WM tracts, the same can’t be said about smaller structures and structurally atypical brains [11, 37]. In TBSS, to overcome the effect of normalization, we measure FA values at the centre of the tract via projection of these values on a skeleton [10], which was revealed to be a valuable tool for DTI investigation. This projection is achieved by searching for the maximum value, assumed to represent the centre, perpendicular to the skeleton structure [13]. However, not all differences in signal are homogenously distributed to the centre of the tracts, and differences between groups can be too subtle to detect with more conservative techniques where only peak voxels are reflected, and can be affected by anatomical shifts, WM structural abnormalities and registration errors [11, 13]. If ROI-specific measurements are carried out following spatial normalization by masking with atlas-derived pre-defined tracts, the estimated tracts are much larger tracts (~1.5 fold increase), compared to native WM tracts through normalization and up sampling, which leads to inclusion of smoothed voxels and partial volume effects. Other approaches to investigating ROIs include fibre tractography, with its main advantage, from the clinical investigations perspective, being the possibility to derive measures from the entire fibre bundle, instead of one of its segments, and as a discovery technique for WM connectivity [38]. However, such measurements can be challenged by the presence of structural abnormalities leading to false positive bundles [39], and different approaches should be used when specific and predefined ROIs are investigated. In a previous study that used the same clinical cohort as part of a larger cohort, individual detection of WM injury in patients with TBI was carried out using TBSS and a subset of seven reliable tracts out of the 47 tracts available [15]. This study also found that patients with focal abnormalities have a higher incidence of WM abnormalities (~40% of cases) than TBI patients with unremarkable standard MRI (~33%). However, with the new pipeline, it is possible to investigate more ROIs without being limited to the seven reliable tracts indicated by this study. It is also possible to include segmentation of specific areas within the ROIs such as the boundaries of the tracts. Because the WM map was eroded to exclude partial volume, the boundaries of the tracts would also reflect the exclusion of the partial volume. However, this application is only possible in tracts that are large enough such as the middle cerebellar peduncle or the corpus callosum.

Using SSDS can enable longitudinal studies of volume changes in WM ROIs over time in both group and individual investigations. SSDS also eliminates the need to manually delineate and mask structural abnormalities (lesions, tumors, etc…), given that the technique relies on a WM map to guide the segmentation; and WM map estimation excludes low-intensity signals from the T1 image [27]. Several steps in the pipeline also curb the issues arising from partial volume, reducing their effect on the results. These include using a T1 WM binary map as an anatomical guide, eroding the WM map, and adding optional erosions of the tracts before and after registering them to native space. It is important to note that the kernel used for erosion only excludes the outermost voxels. The partial volume is therefore limited to the resolution of the diffusion image.

This pipeline can be applied to any measure derived from diffusion images, although we have demonstrated its utility in the analysis of FA data. However, the resolution and performance of the algorithm will ultimately be determined by the resolution and performance of the underlying DWI acquisition. Our approach can either be used independently or to complement group-level analysis such as VBA or TBSS. SSDS also has significant advantages if specific parts of WM tracts need to be sampled, for example WM boundaries or tract-specific boundaries. Such investigations would be of interest for a variety of conditions, including specific subsets of TBI such as blast-related TBI and autism [40, 41]. The main limitations of SSDS relate to its application to smaller tracts, which tend to have higher variation (e.g. the superior Fronto-Occipital Fasciculus and the hippocampal projection of the Cingulum). The effect of noise on FA measures has well been documented [22] and impacts on the variance in anisotropy. This, however, is not exclusive to SSDS, and implies high error rates when using alternative methods. Another limitation would be the performance of registration if and when gradient field maps have not been acquired, or the quality of the structural image segmentation in case abnormalities are present. Future investigations using SSDS will have to determine the minimal angular resolution, spatial resolution, and signal to noise ratio for SSDS to still be able to perform, as well as the applicability in small white matter tracts when using higher spatial resolution diffusion imaging.

## Conclusion

In conclusion, we suggest the use of SSDS for clinical DTI studies where individual segmentations and subject-specific diffusion metrics estimations might be more revealing than group-level analyses. SSDS can therefore replace manual ROIs, is an automated and reliable pipeline, standardized for more homogenous and reproducible methodology across studies.

## Supporting information

S1 Fig Example of segmentation of whole tracts. Tracts are color-coded; Red: splenium of corpus callosum, Orange: body of corpus callosum, Green: genu of corpus callosum, Light Blue: corticospinal left and right, Dark Blue: Middle Cerebellar Peduncle.
(TIF)

S2 Fig Repeatability of measures across visits for boundaries of the WM. 3D correlation plot of mean FA value and number of voxels for all three visits per tract, per subject. Each color represents a region of the boundary of the WM.
(TIF)

## Abbreviations

SSDS: Subject-Specific Diffusion Segmentation
TBSS: Tract-Based Spatial Statistics
TBI: Traumatic Brain Injury
VBA: Voxel-based analysis
ROI: Region of interest
BBR: Boundary-based registration
COV: Coefficient of variation
