# Supplementary figures and images for: Automation and standardization of subject-specific region-of-interest segmentation for investigation of diffusion imaging in clinical populations
Source: PLoS One. 2022 Dec 8;17(12):e0268233. doi: 10.1371/journal.pone.0268233 (PMC9731501; doi:10.1371/journal.pone.0268233)

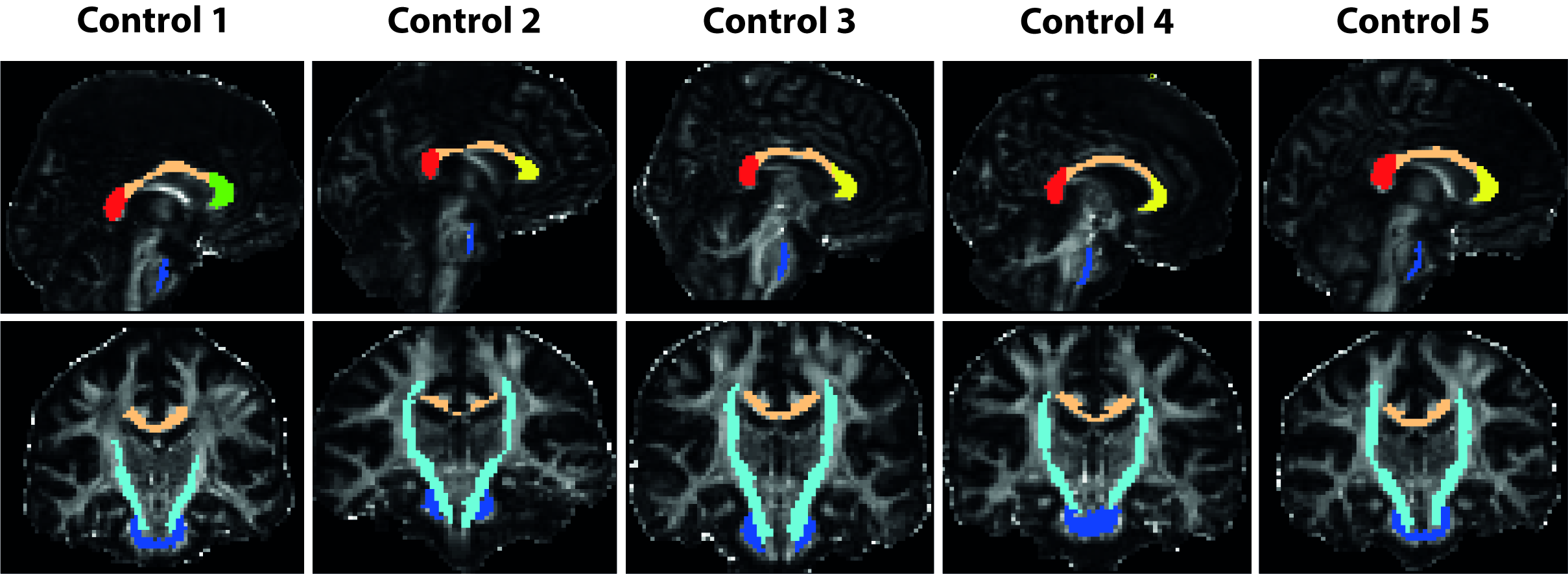

Supplement: S1 Fig — Tracts are color-coded; Red: splenium of corpus callosum, Orange: body of corpus callosum, Green: genu of corpus callosum, Light Blue: corticospinal left and right, Dark Blue: Middle Cerebellar Peduncle. (TIF) [file pone.0268233.s001.tif]

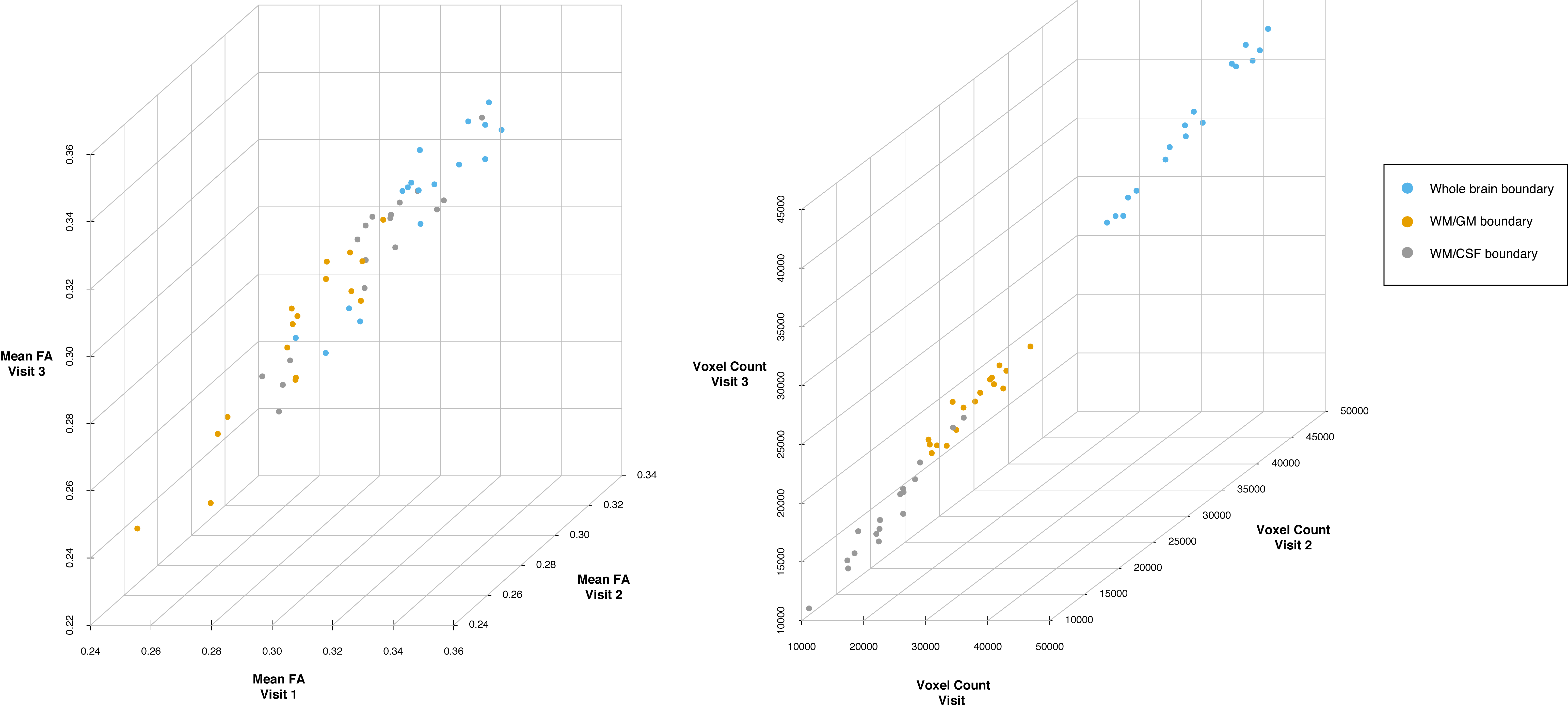

Supplement: S2 Fig — 3D correlation plot of mean FA value and number of voxels for all three visits per tract, per subject. Each color represents a region of the boundary of the WM. (TIF) [file pone.0268233.s002.tif]
